# Genomic Analysis of the Rare Slightly Halophilic Myxobacterium “*Paraliomyxa miuraensis*” SMH-27-4, the Producer of the Antibiotic Miuraenamide A

**DOI:** 10.3390/microorganisms11020371

**Published:** 2023-02-01

**Authors:** Ying Liu, Makoto Ojika

**Affiliations:** Department of Applied Biosciences, Graduate School of Bioagricultural Sciences, Nagoya University, Nagoya 464-8601, Aichi, Japan

**Keywords:** halophilic myxobacteria, *Paraliomyxa miuraensis*, Nannocystaceae, miuraenamide A, biosynthetic gene cluster, draft genome

## Abstract

Halophilic/halotolerant myxobacteria are extremely rare bacteria but an important source of novel bioactive secondary metabolites as drug leads. A slightly halophilic myxobacterium, “*Paraliomyxa miuraensis*” SMH-27-4, the producer of the antifungal antibiotic miuraenamide A, was considered to represent a novel genus. This study aimed to use the whole-genome sequence of this difficult-to-culture bacterium to provide genomic evidence supporting its taxonomy and to explore its potential as a novel secondary metabolite producer and its predicted gene functions. The draft genome was sequenced and de novo assembled into 164 contigs (11.8 Mbp). The 16S rRNA gene sequence-based and genome sequence-based phylogenetic analyses supported that this strain represents a novel genus of the family Nannocystaceae. Seventeen biosynthetic gene clusters (BGCs) were identified, and only five of them show some degree of similarity with the previously annotated BGCs, suggesting the great potential of producing novel secondary metabolites. The comparative genomic analysis within the family Nannocystaceae revealed the distribution of its members’ gene functions. This study unveiled the novel genomic features and potential of the secondary metabolite production of this myxobacterium.

## 1. Introduction

Myxobacteria are famous for their complex life cycle of multicellular fruiting body formation and cooperative preying behavior. They are considered as a template for the study of bacterial social behavior [1,2,3]. Aside from this, their potential for immense secondary metabolite production also makes them candidates for a next-generation microbial drug factory [4,5,6]. Since their first description as a novel taxon in 1892 [7], myxobacteria were considered terrestrial bacteria until 1998, when the first isolation of obligate halophilic marine myxobacteria was reported by Iizuka et al. [8]. Although the difficulty with isolation and cultivation obstructs the discovery of halophiles, the limited number of strains already shows great potential for producing novel bioactive leads with unique molecular scaffolds and activities [9,10,11,12]. To date, all discovered and cultivable halophilic myxobacteria are grouped into the suborder Nannocystineae, which consists of two families, Kofleriaceae and Nannocystaceae [10].

**Figure 1:**
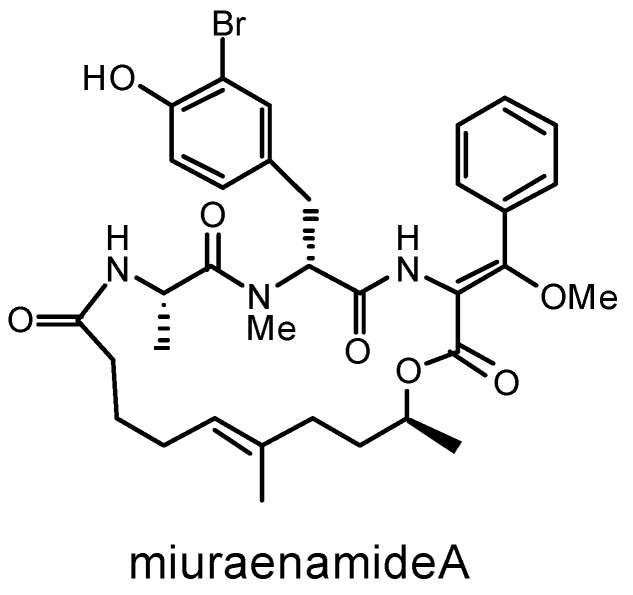
Chemical structure of miuraenamide A.

In 2006, the myxobacterium strain SMH-27-4 was isolated from a near-seashore soil in Japan and tentatively named “*Paraliomyxa miuraensis*” [13]. The phylogenetic analysis based on a partial 16S rRNA gene sequence suggested that it represents a new genus of the family Nannocystaceae [13,14]. Nannocystaceae is the most ecologically diverse myxobacterial family and, besides *Paraliomyxa*, contains four genera: two marine-derived genera *Plesiocystis* and *Enhygromyxa*, a brackish water genus *Pseudenhygromyxa*, and a terrestrial genus *Nannocystis* [15]. In 2016, Iizuka reported the chemotaxonomic and physiological characteristics of the strain SMH-27-4 and provided the descriptions of “*Paraliomyxa*” gen. nov. and “*Paraliomyxa miuraensis*” sp. nov. (Reference S1) [14]. The optimal salt concentrations for the growth of “*P. miuraensis*” SMH-27-4 were determined as 0.5–1.0% (*w*/*v*) NaCl, and requires Mg^2+^ and Ca^2+^ for its growth [13]. On the agar plate, the strain swarms in a radial pattern and sometimes cleaves the agar gel matrix [14]. The major cellular fatty acids are iso-C_15:0_ and iso-C_17:0_. They do not degrade filter papers or grow in a yeast medium, such as VY/2 agar, generally used for terrestrial myxobacteria. The above characteristics were shared with the slightly halophilic myxobacteria *Pseudenhygromyxa* [14,16]. However, its major cellular quinone is MK-8, and long-chain polyunsaturated fatty acids were not detected. These two properties are the same as the terrestrial strain *Nannocystis exedens* DSM 71 [14,17]. No distinct fruiting body was observed for this strain, which made it more enigmatic [13,14]. The major secondary metabolite of “*P. miuraensis*” SMH-27-4, miuraenamide A (Figure 1), exhibited potent antifungal activity, especially against the phytopathogenic oomycete *Phytophthora capsici* at a minimum inhibition dose of 25 ng/disk by inhibiting the mitochondrial respiratory chain [13]. It was also shown to significantly change the morphology of the cytoplasm and nucleus of a tumor cell line by stabilizing actin filaments [18]. Over the last decade, its total synthesis and biological activity as an actin filament stabilizer have been broadly explored [19,20,21,22,23,24,25,26]. The potential of miuraenamide A for medical applications makes this strain more worthy of investigation.

To confirm the taxonomy of “*P. miuraensis*” SMH-27-4 as well as the productivity of other secondary metabolites and the distribution of gene functions, we comprehensively analyzed its draft genome sequence.

## 2. Materials and Methods

### 2.1. Cultivation and Genomic DNA Isolation

Isolation of “*P. miuraensis*” SMH-27-4 was previously described [13]. The strain is registered in DBRP as STAJ0000000110262 and deposited in NBRC (Kisarazu, Chiba, Japan) as NBRC 111985. On the VY/2-1/5SWS agar plate [13], the strain swarms in a radial pattern and burrows into the agar. The outer edge of the swarm was cut out of the agar strip and frozen as glycerol stock in 12% (*w*/*v*) glycerol solution at −80 °C [14]. After thawing, the glycerol stock was washed with autoclaved Milli-Q water and planted on the Vy_5_.S_75.15_ agar plate (see below). The plates were cultured for 14 days at 27 °C. Ten 0.5 cm square agar strips were cut out of the swarm edge and added to 750 mL of N_2.0_-S_75.10_ broth (see below) in a 2 L Erlenmeyer flask, which was shaken at 180 rpm. The cells tended to aggregate together, forming cell masses in the liquid broth. After 14 days of cultivation, the broth was filtrated by suction, and the orange or yellow cell mass on the filter paper was collected. Approximately 50 mg (wet weight) of the cells was used for the isolation of genomic DNA by a QIAGEN Genomic-tip 100/G (QIAGEN, Venlo, Nederland) and Genomic DNA Buffer Set (QIAGEN) according to standard protocols. 

Vy_5_.S_75.15_ medium: 0.5% (*w*/*v*) yeast cake, 0.01% (*w*/*v*) Bacto™ yeast extract (Thermo Fisher Scientific, Waltham, MA, USA) and 1.5% (*w*/*v*) NaCl were suspended in 75% Sea Water Solution (SWS) [8], and pH was adjusted to 7.2–7.4 with 1 M NaOH before autoclaving. Vitamin B_12_ (0.5 mg/1 mL water) was sterilized by filtration and added to the autoclaved solution (1 L). Yeast cake: Dried yeast (Mitsubishi Tanabe Pharma, Osaka, Japan) was suspended in Milli-Q water (10% (*w*/*v*)). The supernatant was discarded after centrifugation (10,000× *g* rpm, 5 min), and the yeast cake was washed three times with water by suspending, vortexing, and centrifugation. The cake was stored at −30 °C until use. N_2.0_-S_75.10_ medium: 2% (*w*/*v*) casein sodium (FUJIFILM Wako Pure Chemical Corporation, Osaka, Japan) and 1% (*w*/*v*) NaCl were suspended in 75% Sea Water Solution (SWS), and the pH was adjusted to 7.2–7.4 with 1 M NaOH before autoclaving. Vitamin B_12_ (0.5 mg/1 mL water) was sterilized by filtration and added to the autoclaved solution (1 L).

### 2.2. Draft Genome Sequencing, Assembly, and Annotation

The whole genome was sequenced using the Illumina HiSeq platform, paired-end, 101 bp X 2 sequencing. The adapter sequence and low-quality bases were trimmed from raw reads files using Cutadapt (version 1.1) [27] and Trimmomatic (version 0.32) [28]. After trimming, sequence reads were assembled into contigs using the de novo genome assembler Velvet (version 1.2.08) [29]. The gapclose module of Platanus (version 1.2.1) [30] was then applied to reduce the N content introduced into the genome during scaffolding. The contigs shorter than 200 bp were removed. Automated annotation of the draft genome sequence was performed with the prokaryotic genome annotation pipeline (PGAP) of NCBI (version 6.3) [31]. The estimated genome size was calculated by KmerGenie (version 1.7051) based on Kmer analysis. The completeness of genome assembly and annotation were assessed using benchmarking universal single-copy orthologs (BUSCO) scores (version 5.3.2) [32].

### 2.3. Phylogenetic Analysis

The complete 16S rRNA gene sequence was identified by PGAP genome annotation. A dataset of 16S rRNA gene sequences of 52 *Myxococcales* strains and *Desulfovibrio desulfuricans* Essex 6 were retrieved from GenBank (Table S1). *D. desulfuricans* Essex 6 was used as an outgroup to root the tree. The 16S rRNA gene sequences were aligned by MAFFT (version 7.487) [33]. Maximum likelihood analyses were constructed in IQ-TREE (version 2.1.4-beta) [34] using the best-fit model TN + F + R4, selected by the software according to the Bayesian information criterion (BIC) scores. Bootstrap values were based on 1000 replicates, and the obtained tree was visualized using iTOL [35].

The genome sequences of 51 strains of the order Myxococcales were retrieved from the NCBI reference sequence (RefSeq) database (Table S2). The average nucleotide identity (ANI)-based distance tree was produced using the ANI-matrix genome distance calculator [36]. The ANI tree was clustered using the neighbor-joining method.

### 2.4. BGCs Prediction and Generation of Similarity Networks

The draft genome sequence of “*P. miuraensis*” SMH-27-4 was mined using the antibiotics and secondary metabolite analysis shell (antiSMASH) (version 6.1.1) [37] to identify the secondary metabolite protoclusters using the “strict” setting. A machine learning-based ribosomally synthesized and post-translationally modified peptide (RiPPs)-mining tool, RiPPMiner [38] was also used to retrieve the RiPP protoclusters. The protoclusters were regarded as BGCs under the following criteria: (1) in the case that the overlapping area of two close protoclusters contains core biosynthetic genes, those with different cluster types are united to one “hybrid” BGC, whereas those with same cluster types are united to one BGC; (2) the following protoclusters predicted by antiSMASH were not considered as BGCs: “RiPP-like”, non-specific RiPP-containing post-translational modification proteins such as DUF 692 family, a function-unknown protein family, and “other” protoclusters that cannot be classified into the known categories. All the predicted BGCs were then analyzed using the biosynthetic gene similarity clustering and prospecting engine (BiG-SCAPE) software package (version 1.1.2) [39], with the MiBIG database (version 2.1) [40] as a reference. BiG-SCAPE was conducted on auto mode with a distance cut-off score of 0.75 and the parameters of “--clans-off”, “--no_classify”, and “--mix”. The “*P. miuraensis*” SMH-27-4 BGCs-related records were extracted, and the generated networks were visualized with Cytoscape (version 3.8.2) [41].

### 2.5. Identification of Orthologous Proteins and Functional Categorization 

The genome sequences of four strains of the family Nannocystaceae (*Nannocystis exedens* DSM 71, *Pseudenhygromyxa* sp. WMMC2535 (GCA_011083025.2), *Plesiocystis pacifica* SIR-1, and *Enhygromyxa salina* DSM 15201 (GCA_000737335.3)) were retrieved from the NCBI genome and RefSeq database. Their genomes, including that of “*P. miuraensis*” SMH-27-4, were re-annotated by the stand-alone PGAP (version 2021-07-01. build5508) with the same parameters. The orthologous proteins were identified by Proteinortho [42]. The protein functional annotation was conducted using the domain-based annotation tool reCOGnizer (version 1.7.2) [43]. The results derived from the clusters of orthologous groups of proteins (COGs) database were used for functional categorization, which consists of 26 categories. Some proteins were classified into more than one functional category. In this case, each protein was assigned as an equal portion of weight for each functional category.

### 2.6. Availability of Nucleotide Sequence Data

The whole genome shotgun project of “*P. miuraensis*” SMH-27-4 has been deposited at DDBJ/ENA/GenBank under the accession number JAOVZF000000000. The version described in this paper is version JAOVZF010000000. The raw sequencing data were submitted to the Sequence Read Archive database under the accession number SRR21887204.

## 3. Results

### 3.1. Draft Genome Sequencing, Assembly, and Annotation 

A total of 2313 Mbp was obtained from Illumina HiSeq paired-end sequencing. The results are summarized in Table 1. The assembled “*P. miuraensis*” SMH-27-4 draft genome size is 11,849,290 bp, equal to 100.1% of the estimated genome size based on Kmer analysis. The overall GC content is 69.7%. The draft genome consists of 164 contigs, with the N50 and L50 values of 398,768 bp and 11, respectively. The completeness of the draft genome assembly was evaluated by calculating coverage for a set of single-copy orthologous genes in deltaproteobacteria using BUSCO. The results showed that the genome coverage rate was 93.0 %. PGAP annotation predicted 9280 genes in the genome, including 31 pseudogenes, 84 RNAs, and 9156 protein-coding genes that account for 90.7% coding density. The 9156 protein sequences were aligned to the BUSCO database to evaluate the annotation quality, and the coverage rate of 92.6% suggested a high degree of completeness of the gene prediction. A total of 38.3% (3508) of the protein-coding genes were annotated as hypothetical proteins. 

### 3.2. Phylogenetic Analysis

**Figure 2:**
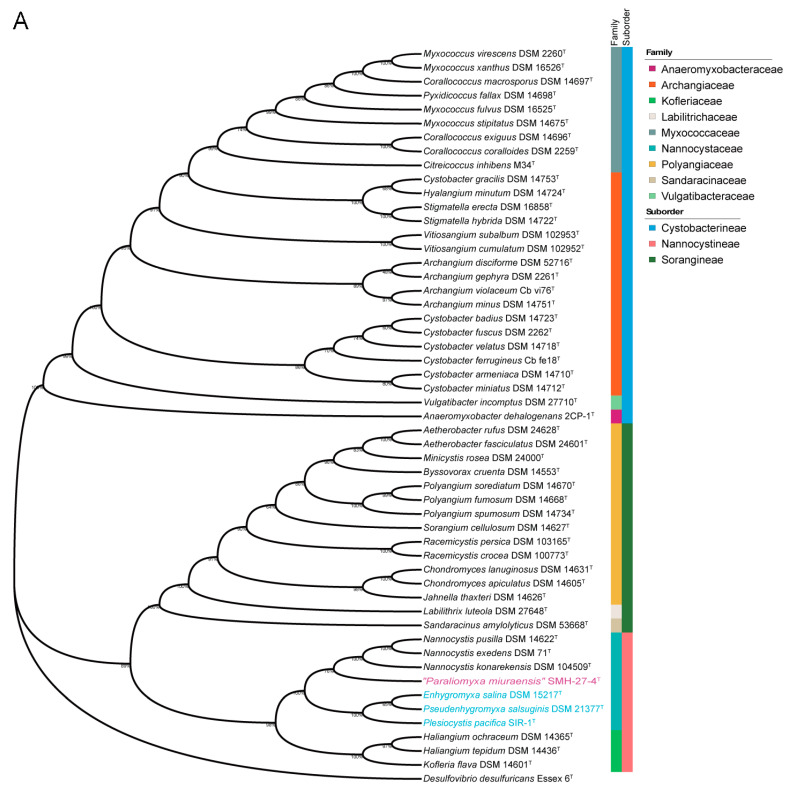

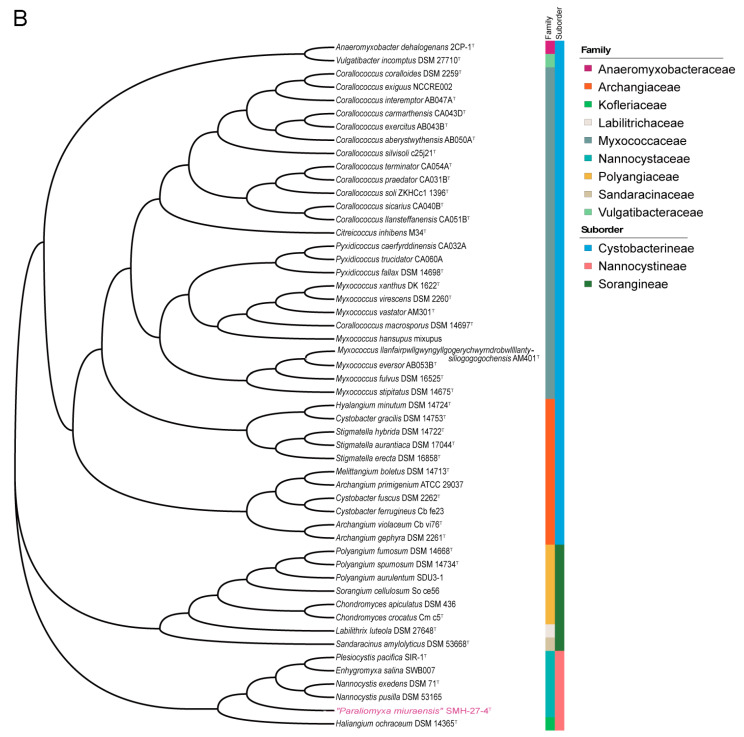
Phylogenetic trees of myxobacteria in comparison with “*P. miuraensis*” SMH-27-4. (**A**) 16S rRNA gene sequence-based tree. The numbers at the nodes indicate branch support values. The (slightly) halophilic strains of the family Nannocystaceae are in light blue. (**B**) Genome sequence-based tree.

Both the 16S rRNA gene sequence-based and genome sequence-based phylogenetic analyses of the order Myxococcales were performed. The complete 16S rRNA gene sequence of “*P. miuraensis*” SMH-27-4 consisted of 1544 bp (locus_tag = OEB96_36580), which is identical to the reported partial 16S rRNA gene sequence (1504 bp) [13]. In the 16S rRNA gene sequence-based phylogenetic tree (Figure 2A), the strains in the family Nannocystaceae were divided into two subclades with a support value of 100%. Although “*P. miuraensis*” SMH-27-4 shared a subclade with the terrestrial genus *Nannocystis*, it branched out of the strains of the genus *Nannocystis* with a support value of 76%. The strains of (slightly) halophilic genera (*E. salina* DSM 15217, *P. salsuginis* DSM 21377, and *P. pacifica* DSM 14875) formed the other subclade.

To perform genome-based taxonomic classification, the average nucleotide identity (ANI) was compared between “*P. miuraensis*” SMH-27-4 and 51 other sequenced myxobacterial strains. The resulting genome-based phylogenetic tree (ANI tree, Figure 2B) indicated that “*P. miuraensis*” SMH-27-4 was grouped into the family Nannocystaceae but did not form any subclade with other strains of this family. This result is similar to the 16S rRNA gene sequence-based tree (Figure 2A), supporting the novelty of the genus “*Paraliomyxa*”. 

### 3.3. Biosynthetic Gene Clusters (BGCs)

**Figure 3:**
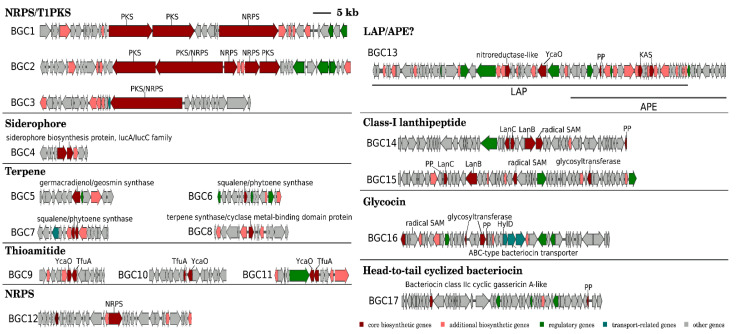
Organizations of putative BGCs in the genome of “*P. miuraensis*” SMH-27-4.

The antiSMASH detected 30 protoclusters, which, due to the presence of some hybrid types, were partially compiled to obtain 24 candidate BGC regions for secondary metabolite biosynthesis (Table S3). The RiPPMiner retrieved five ribosomally synthesized and post-translationally modified peptide (RiPP) protoclusters (Table S4). After the removal of protoclusters of unspecific BGC type and combination of close protoclusters based on the rules described in Section 2.4, 17 BGCs in total were annotated from the genome of “*P. miuraensis*” SMH-27-4 (Figure 3 and Figure S1). The BGCs include three hybrids of non-ribosomal peptide synthetases/type I polyketide synthase (NRPS/T1PKS), one siderophore, four terpenes, three thioamitides, one NRPS, one linear azol(in)e-containing peptide/aryl polyene hybrid (LAP/APE, probably hybrid), two class-I lanthipeptides, one glycocin and one head-to-tail cyclized bacteriocin. BGC1 (NRPS/T1PKS) was regarded as the BGC for miuraenamide A because the predicted substrate selectivity of its eight modules and their assembly order matched the backbone of miuraenamide A, consisting of five polyketide units and a tripeptide composed of alanine, tyrosine, and phenylalanine. The biosynthesis mechanism of miuraenamide A will be validated in future research.

**Figure 4:**
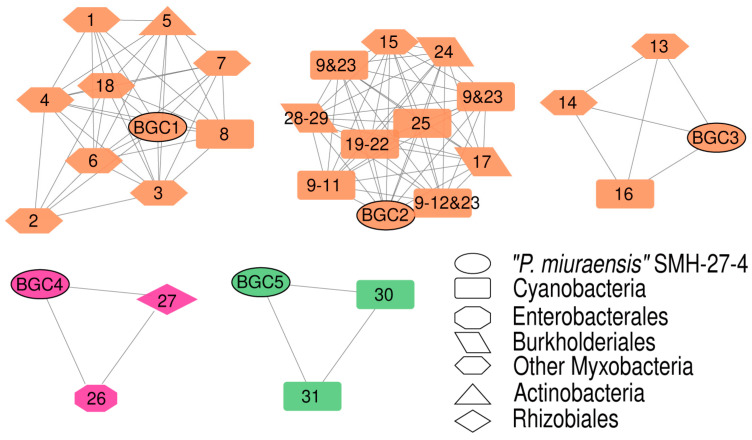
BGC sequence similarity networks of “*P. miuraensis*” SMH-27-4 and related sequences from the MIBiG database. Nodes are color-coded according to BGC types and shape-coded according to biosynthesis origins (legend). Numbered nodes represent the BGCs from the MIBiG database. The chemical structures of the products encoded by them were listed with the same numbers in Figure S2.

The BGC sequence similarity network obtained by Big-SCAPE suggested the great potential of this strain to produce novel secondary metabolites. Each of the five BGCs (BGC1–BGC5) formed a cluster with the known BGCs from the minimum information about a biosynthetic gene cluster (MIBiG) database (Figure 4 and Figure S2), while the other 12 BGCs showed no similarity with the BGCs for known products. The detailed gene organizations of the BGCs are shown in Figures S3–S7. BGC1 was clustered with the BGCs encoding the biosynthesis of nine myxobacterial depsipeptides: chondramide A (1), epothilone (2), nannocystin A (3), myxothiazol (4), microsclerodermin (6), thaxteramide C (7), antalid (18), and two cyclic depsipeptides from actinobacteria and cyanobacteria, microtermolide A (5) and nodularin (8). BGC2 was clustered with the BGCs encoding the following metabolites’ biosynthesis: two groups of cyanobacterial cyclic lipopeptides minutissamides (9–12) and puwainaphycins (19–23), a cyanobacterial non-ribosomal peptide nostopeptolide A2 (25) [44], the membrane morphology-disrupting fungicide occidiofungin A (17) [45], the fungal chlamydospore formation-inducer ralsolamycin (24) [46], the delftibactins that are able to detoxify toxic soluble gold (28, 29) [47], and the myxobacterial linear peptide myxoprincomide-c506 (15). BGC3 was clustered with the BGCs encoding the biosynthesis of the myxobacterial cyclic depsipeptides myxochromides (13, 14) and the antialgal cyanobacterial peptide kasumigamide (16) [48]. BGC4 was clustered with the BGCs encoding the biosynthesis of the bacterial siderophores aerobactin (26) and ochrobactin (27). BGC5 was clustered with the BGCs encoding the biosynthesis of geosmin (30) and 2-methylisoborneol (31), both of which are responsible for the earthy–musty odor in water.

### 3.4. Distribution of Gene Fuctions of the Strains of the Family Nannocystaceae 

The family Nannocystaceae that contains “*P. miuraensis*” SMH-27-4 is known to be ecologically diverse. To explore the distribution of the gene functions of this family, a comparative genomic analysis was performed for the genomes of representative strains from five different genera in the family Nannocystaceae: “*P. miuraensis*” SMH-27-4, *N. exedens* DSM 71, *Pseudenhygromyxa* sp. WMMC2535, *P. pacifica* DSM 14875, and *E. salina* DSM 15201. The orthologous protein-coding genes were identified, and the genomic functional annotation was performed by the clusters of orthologous groups (COG) approach. Here, the protein-coding genes were divided into core, accessory, and strain-specific genes based on the distribution of orthologous genes through the examined strains (Table 2). The core genes, common genes in the five strains, accounted for 16–19% of each genome, while the strain-specific genes accounted for 44–59%. The others were classified into the accessory genes that occupied 25–37% of the genomes. The COG approach performs microbial genome-wide functional annotation against the COG database, which consists of COGs with manually curated annotation and classifies the COGs into 26 functional categories. The distribution of COG functional categories can reveal to some extent the metabolic or physiological features of the bacteria. Approximately 43% of the protein-coding genes of each strain were classified in COG superfamilies, while more than half were not, suggesting that the genomic resources of myxobacteria are underexplored. The numbers of genes of each COG functional category are listed in Table S5.

**Figure 5:**
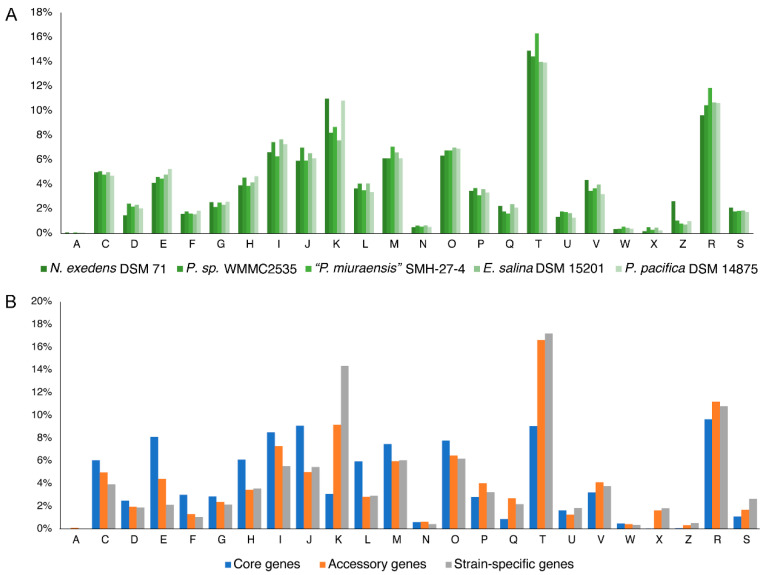
Distribution of COG functional categories in five strains of the family Nannocystaceae. (**A**) Distribution of functional categories in COGs by strains; (**B**) Average distribution of functional categories of all strains by the core, accessory, and strain-specific genes. Abbreviations: A: RNA processing and modification; C: Energy production and conversion; D: Cell cycle control, cell division, chromosome partitioning; E: Amino acid transport and metabolism; F: Nucleotide transport and metabolism; G: Carbohydrate transport and metabolism; H: Coenzyme transport and metabolism; I: Lipid transport and metabolism; J: Translation, ribosomal structure and bio-genesis; K: Transcription; L: Replication, recombination and repair; M: Cell wall/membrane/envelop biogenesis; N: Cell motility; O: Posttranslational modification, protein turnover, chaperones; P: Inorganic ion transport and metabolism; Q: Secondary metabolites biosynthesis, transport and catabolism; T: Signal transduction mechanisms; U: Intracellular trafficking, secretion, and vesicular transport; V: Defense mechanisms; W: Extracellular structures; X: Mobilome: prophages, transposons; Z: Cytoskeleton; R: General function prediction only; S: Function unknown.

The distribution of functional categories of all COGs was similar between the five strains (Figure 5A), indicating a possibility of conservation in the genomic functions within this family. Except for the poorly characterized categories [R] and [S], the most abundant categories were related to signal transduction mechanisms [T], transcription [K], and lipid transport and metabolism [I]. The abundance of these categories suggests that these myxobacterial strains evolved particular environmental response mechanisms of extra- and intracellular signals that include diverse proteins and metabolites. The average population of functional categories in this family quite varied by the number of core, accessory, and strain-specific genes (Figure 5B). In the accessory genes, signal transduction mechanisms [T] showed the significantly high distribution (16.6%) compared to the other categories (lower than 11.2%). In the strain-specific genes, both signal transduction mechanisms [T] and transcription [K] categories accounted for high distributions (14.4–17.2%). On the other hand, the distribution of the core genes was more homogeneous. This trend was evidenced by the standard deviations of the distribution of 4.3%, 3.9%, and 3.4% for the strain-specific, accessory, and core genes, respectively. The variation of the gene functions and the percentage of the gene number (Table 2) in the strain-specific genes give an account of the diversity of these genera in the family Nannocystaceae.

## 4. Discussion

The draft genome of “*P. miuraensis*” SMH-27-4 was sequenced, and de novo assembled into 11.8 Mbp of 164 contigs. Both Kmer and BUSCO analyses suggested a high degree of completeness of the genome assembly. We did not use third-generation long-read sequencing technologies but acknowledge that they facilitate the de novo assembly of complete genomes, as longer reads can be aligned to repetitive sequences with high confidence and increase assembly contiguity. Although the Illumina-based secondary generation short-read sequencing can assemble over 99% of the complete genome, the few missing parts may include essential genes, as recently illustrated in *Pseudomonas aeruginosa* [49]. The results of the 16S rRNA gene-sequence-based phylogenetic analysis and the genome-based taxonomic classification by ANI values were consistent with each other and indicated that this difficult-to-culture myxobacterium represents a novel genus in the family Nannocystaceae. Aside from the BGC for miuraenamide A, the strain has 16 other BGCs that showed low or no similarity with the BGCs for known products, revealing a great potential of the strain to produce novel secondary metabolites. The similar distribution of the COG functional categories through the strains from five genera within the family Nannocystaceae indicated conserved genomic functions of this family. On the other hand, the average distribution of COG functional categories by the core, accessory, and strain-specific genes suggests that the five genera have diverse signal transduction and gene transcription mechanisms. Regardless of the taxonomic and physiological novelty of this rare slightly halophilic myxobacterium, the potential of great secondary metabolites production makes it worthy of studying. 

## 5. Conclusions

Myxobacteria are common in terrestrial habitats and known for their potential to produce novel natural products, whereas marine-derived (or halophilic) ones are quite rare and only seven species (five genera) have been identified since the isolation of the first marine myxobacteria *H. ochraceum* and *P. pacifica* in 1998 [8]. Although these marine myxobacteria are regarded as a good factory of valuable secondary metabolites beyond the terrestrial ones, their cultivation is generally difficult and takes a long period for enough growth. Their genomic information is therefore important to elucidate their great potential to produce novel leads with unique molecular scaffolds and bioactivities. “*P. miuraensis*” SMH-27-4 produces a series of PKS/NRPS hybrid molecules named miuraenamides [13], but its metabolic profile indicated a scarcity of metabolite diversity; no other distinct metabolites were detected in the extracts. The genomic analysis of this strain was therefore performed in this study and revealed the presence of 17 BGCs for producing metabolites, one of which was estimated to encode the biosynthesis of miuraenamides. The complete genome sequence was not available in this study due to the extremely difficult cultivation and DNA extraction from aggregated mucous cells. Nevertheless, because of the high-quality sequence data, 93% coverage of the complete genome (the rest could be repetition), and no overlooking of other possible BGCs, the present draft genome information could contribute to improving the inadequate expertise in the marine myxobacterial genomic functions, especially for hidden biosynthetic machineries leading to brand-new natural products. Further studies will be needed to reveal the mechanism of the miuraenamide biosynthesis as well as more precise genomic analysis. 

## Figures and Tables

**Table 1:** Assembly and annotation statistics for the draft genome sequence of “*P. miuraensis*” SMH-27-4.

Number of contigs	164
GC Content (%)	69.7
Estimated genome size based on Kmer analysis	11,832,550 bp
Assembled genome size	11,849,290 bp (100.1%)
N50 (bp)	398,768
L50	11
Genes (total)	9280
Pseudogenes (total)	40
Genes (RNA)	84
tRNAs	77
rRNAs	1, 1, 1 (5S, 16S, 23S)
ncRNAs	4
Genes (coding)	9156
Coding density	90.7%
Hypothetic proteins	3508 (38.3%)
Percentage (%) of complete BUSCOs in the genome assembly	93.0%
Percentage (%) of complete BUSCOs among the annotated genes	92.6%

**Table 2:** Comparative genomic analysis of five strains of the family Nannocystaceae.

Strains	Protein-Coding Genes	Orthologous Genes			COG Annotated Genes
		Core Genes	Accessory Genes	Strain-Specific Genes	
*Pm*	9145 (100%)	1460 (16%)	2500 (27%)	5185 (57%)	3891 (43%)
*Ne*	9295 (100%)	1469 (16%)	2347 (25%)	5479 (59%)	4020 (43%)
*Ps*	7726 (100%)	1475 (19%)	2847 (37%)	3404 (44%)	3389 (44%)
*Pp*	8182 (100%)	1448 (18%)	2859 (35%)	3875 (47%)	3516 (43%)
*Es*	8079 (100%)	1448 (18%)	2788 (35%)	3843 (48%)	3390 (42%)

The strains included in this analysis are the following: *Pm* “*P. miuraensis*” SMH-27-4, *Ne* *N. exedens* DSM 71, *Ps* *Pseudenhygromyxa* sp. WMMC2535, *Pp* *P. pacifica* SIR-1, *Es* *E. salina* DSM 1520.

## Data Availability

The data presented in this study are available in Supplementary Material and via accession numbers described in the Section 2 of this article.

## Supplementary Materials

The following supporting information can be downloaded at: https://www.mdpi.com/article/10.3390/microorganisms11020371/s1, Table S1: Information of 16S rRNA genes used in Figure 2A; Table S2: Information of genome sequences used in Figure 2B; Table S3: Candidate secondary metabolite BGC regions identified with antiSMASH; Table S4: RiPP protoclusters revealed by RiPPMiner; Table S5: COG classification of the protein-coding genes of five strains in the family Nannocystaceae; Figure S1: Locations of BGCs. Figure S2: Chemical structures of the secondary metabolites described in Figure 4; Figure S3: Organizations of BGC1 and its related BGCs of other species; Figure S4: Organizations of BGC2 and its related BGCs of other species; Figure S5: Organizations of BGC3 and its related BGCs of other species; Figure S6: Organizations of BGC4 and its related BGCs of other species; Figure S7: Organizations of BGC5 and its related BGCs of other species; Reference S1: Description of *Paraliomyxa* gen. nov./*Paraliomyxa miuraensis* sp. nov. (a part of Ref. [14]).
