# Supplementary material for: Genomic Analysis of the Rare Slightly Halophilic Myxobacterium “Paraliomyxa miuraensis” SMH-27-4, the Producer of the Antibiotic Miuraenamide A
Source: Microorganisms. 2023 Feb 1;11(2):371. doi: 10.3390/microorganisms11020371 (PMC9960870; doi:10.3390/microorganisms11020371)
Supplement: Supplementary file 1 [file microorganisms-11-00371-s001.zip › microorganisms-2034915-supplementary.pdf]

Supplementary Materials

Genomic Analysis of the Rare Slightly Halophilic Myxobacterium "*Paraliomyxa miuraensis*" SMH-27-4, the Producer of the Antibiotic Miuraenamide A

Ying Liu and Makoto Ojika*

Department of Applied Biosciences, Graduate School of Bioagricultural Sciences, Nagoya University, Nagoya 464-8601, Aichi, Japan

Contents

Table S1. Information of 16S rRNA genes used in Figure 2A.
Table S2. Information of genome sequences used in Figure 2B.
Table S3. Candidate secondary metabolite BGC regions identified with antiSMASH.
Table S4. RiPP protoclusters revealed by RiPPMiner.
Table S5. COG classification of the protein-coding genes of five strains in the family Nannocystaceae.
Figure S1. Locations of BGCs.
Figure S2. Chemical structures of the secondary metabolites described in Figure 4.
Figure S3. Organization of BGC1 and its related BGCs of other species.
Figure S4. Organization of BGC2 and its related BGCs of other species.
Figure S5. Organization of BGC3 and its related BGCs of other species.
Figure S6. Organization of BGC4 and its related BGCs of other species.
Figure S7. Organization of BGC5 and its related BGCs of other species.
Reference S1. Description of *Paraliomyxa* gen. nov./*Paraliomyxa miuraensis* sp. nov. (a part of ref. 14).

Table S1. Information of 16S rRNA genes used in Figure 2A .

Strain	Accession number of 16S rRNA gene	Length
Haliangium tepidum DSM 14436^{T}	NR_024781.1	1508
Plesiocystis pacifica SIR-1^{T}	NR_024795.1	1484
Enhygromyxa salina DSM 15217^{T}	NR_024807.1	1480
Cystobacter ferrugineus Cb fe18^{T}	NR_025343.1	1517
Chondromyces apiculatus DSM 14605^{T}	NR_025344.1	1534
Chondromyces lanuginosus DSM 14631^{T}	NR_025345.1	1535
Anaeromyxobacter dehalogenans 2CP-1^{T}	NR_027547.1	1545
Nannocystis exedens DSM 71^{T}	NR_040928.1	1480
Kofleria flava DSM 14601^{T}	NR_041981.1	1498
Corallococcus exiguus DSM 14696^{T}	NR_042330.1	1517
Byssovorax cruenta DSM 14553^{T}	NR_042341.1	1529
Stigmatella erecta DSM 16858^{T}	NR_042398.1	1534
Cystobacter armeniaca DSM 14710^{T}	NR_043939.1	1491
Cystobacter badius DSM 14723^{T}	NR_043940.1	1491
Cystobacter fuscus DSM 2262^{T}	NR_043941.1	1491
Cystobacter miniatus DSM 14712^{T}	NR_043942.1	1491
Archangium violaceum Cb vi76^{T}	NR_043943.1	1489
Cystobacter velatus DSM 14718^{T}	NR_043944.1	1491
Pyxidicoccus fallax DSM 14698^{T}	NR_043948.1	1493
Hyalangium minutum DSM 14724^{T}	NR_043949.1	1491
Stigmatella hybrida DSM 14722^{T}	NR_043952.1	1502
Corallococcus coralloides DSM 2259^{T}	NR_074852.2	1536
Haliangium ochraceum DSM 14365^{T}	NR_074917.1	1544
Myxococcus stipitatus DSM 14675^{T}	NR_102512.2	1535
Nannocystis pusilla DSM 14622^{T}	NR_104789.1	1518
Desulfovibrio desulfuricans Essex 6^{T}	NR_104990.1	1542
Myxococcus xanthus DSM 16526^{T}	NR_112544.1	1463
Myxococcus fulvus DSM 16525^{T}	NR_112545.1	1463
Myxococcus virescens DSM 2260^{T}	NR_112546.1	1463
Pseudenhygromyxa salsuginis DSM 21377^{T}	NR_113269.1	1494
Cystobacter gracilis DSM 14753^{T}	NR_115862.1	1491
Corallococcus macrosporus DSM 14697^{T}	NR_115865.1	1493
Sorangium cellulosum DSM 14627^{T}	NR_116678.1	1553
Archangium gephyra DSM 2261^{T}	NR_117459.1	1482
Jahnella thaxteri DSM 14626^{T}	NR_117461.1	1550
Archangium disciforme DSM 52716^{T}	NR_117600.1	1528
Sandaracinus amylolyticus DSM 53668^{T}	NR_118001.1	1506
Archangium minus DSM 14751^{T}	NR_125514.1	1491
Vulgatibacter incomptus DSM 27710^{T}	NR_126181.1	1479
Labilithrix luteola DSM 27648^{T}	NR_126182.1	1483
Minicystis rosea DSM 24000^{T}	NR_134090.1	1550
Aetherobacter fasciculatus DSM 24601^{T}	NR_148644.1	1548
Aetherobacter rufus DSM 24628^{T}	NR_148645.1	1551

Racemicystis crocea DSM 100773 T	NR_149306.1	1549
Racemicystis persica DSM 103165 T	NR_156102.1	1505
Vitiosangium cumulatum DSM 102952 T	NR_156939.1	1526
Vitiosangium subalbum DSM 102953 T	NR_156940.1	1528
Nannocystis konarekensis DSM 104509 T	NR_159914.1	1499
Polyangium fumosum DSM 14668 T	NR_160522.1	1550
Polyangium sorediatum DSM 14670 T	NR_160536.1	1552
Polyangium spumosum DSM 14734 T	NR_160537.1	1553
Citreicoccus inhibens M34 T	NR_173636.1	1536

Table S2. Information of genome sequences used in Figure 2B.

Strain	Assembly accession
Anaeromyxobacter dehalogenans 2CP-1T	GCF_000022145.1
Archangium gephyra DSM 2261T	GCF_001027285.1
Archangium primigenium ATCC 29037	GCF_016904885.1
Archangium violaceum Cb vi76T	GCF_000733295.1
Chondromyces apiculatus DSM 436	GCF_000601485.1
Chondromyces crocatus Cm c5T	GCF_001189295.1
Citreicoccus inhibens M34T	GCF_019039035.1
Corallococcus aberystwythensis AB050AT	GCF_003612165.1
Corallococcus carmarthensis CA043DT	GCF_003611695.1
Corallococcus coralloides DSM 2259T	GCF_000255295.1
Corallococcus exercitus AB043BT	GCF_013116705.1
Corallococcus exiguous NCCRE002	GCF_017302975.1
Corallococcus interemptor AB047AT	GCF_003668875.1
Corallococcus llansteffanensis CA051BT	GCF_003612055.1
Corallococcus macrosporus DSM 14697T	GCF_002305895.1
Corallococcus praedator CA031BT	GCF_003612125.1
Corallococcus sicarius CA040BT	GCF_003611735.1
Corallococcus silvisoli c25j21T	GCF_009909145.1
Corallococcus soli ZKHCc1 1396T	GCF_014930455.1
Corallococcus terminator CA054AT	GCF_003611635.1
Cystobacter ferrugineus Cbfe23	GCF_001887355.1
Cystobacter fuscus DSM 2262T	GCF_000335475.2
Cystobacter gracilis DSM 14753T	GCF_020103725.1
Enhygromyxa salina SWB007	GCF_002994635.1
Haliangium ochraceum DSM 14365T	GCF_000024805.1
Hyalangium minutum DSM 14724T	GCF_000737315.1
Labilithrix luteola DSM 27648T	GCF_001263205.1
Melittangium boletus DSM 14713T	GCF_002305855.1
*Myxococcus eversor*AB053BT	GCF_010894455.1
*Myxococcus fulvus*DSM 16525T	GCF_900111765.1
Myxococcus hansupus mixupus	GCF_000280925.3
Myxococcus llanfairpwllgwyngyllgogerychwyrndrobwllllantysiliogogogochensis AM401T	GCF_006636215.1

Myxococcus stipitatus DSM 14675T	GCF_000331735.1
Myxococcus vastator AM301T	GCF_010894475.1
Myxococcus virescens DSM 2260T	GCF_900101905.1
Myxococcus xanthus DK 1622T	GCF_000012685.1
Nannocystis exedens DSM 71T	GCF_002343915.1
Nannocystis pusilla DSM 53165T	GCF_020073745.1
Plesiocystis pacifica SIR-1T	GCF_000170895.1
Polyangium aurulentum SDU3-1	GCF_005144635.2
Polyangium fumosum DSM 14668T	GCF_005144585.1
Polyangium spumosum DSM 14734T	GCF_009649845.1
Pyxidicoccus caerfyrddinensis CA032A	GCF_010894405.1
Pyxidicoccus fallax DSM 14698T	GCF_012933655.1
Pyxidicoccus trucidator CA060A	GCF_010894435.1
Sandaracinus amylolyticus DSM 53668T	GCF_000737325.1
Sorangium cellulosum So ce56	GCF_000067165.1
Stigmatella aurantiaca DSM 17044T	GCF_900109545.1
Stigmatella erecta DSM 16858T	GCF_900111745.1
Stigmatella hybrida DSM 14722T	GCF_020103775.1
Vulgatibacter incomptus DSM 27710T	GCF_001263175.1

Table S3. Candidate secondary metabolite BGC regions identified with antiSMASH.

Region	Locus (contigs)	Type	From	To
Region 1	JAOVZF010000162.1	T1PKS, NRPS	272536	360088
Region 2	JAOVZF010000163.1	NRPS, T1PKS	507693	593915
Region 3	JAOVZF010000152.1	NRPS, T1PKS	276778	336871
Region 4	JAOVZF010000134.1	siderophore	46935	61490
Region 5	JAOVZF010000149.1	terpene	186818	209109
Region 6	JAOVZF010000161.1	terpene	243759	261662
Region 7	JAOVZF010000157.1	terpene	74778	96691
Region 8	JAOVZF010000161.1	terpene	376547	398295
Region 9	JAOVZF010000163.1	RiPP-like	304798	312818
Region 10	JAOVZF010000163.1	RiPP-like	470102	481025
Region 11	JAOVZF010000162.1	RiPP-like	203558	223128
Region 12	JAOVZF010000157.1	RiPP-like	162301	173182
Region 13	JAOVZF010000133.1	RiPP-like	94703	104650
Region 14	JAOVZF010000160.1	RiPP-like	84202	96013
Region 15	JAOVZF010000139.1	RiPP-like	7088	18050
Region 16	JAOVZF010000158.1	RiPP-like	403850	414788
Region 17	JAOVZF010000145.1	thioamitides	21637	44219
Region 18	JAOVZF010000139.1	thioamitides	85971	108523
Region 19	JAOVZF010000155.1	thioamitides	363283	385917
Region 20	JAOVZF010000159.1	other	55245	97845
Region 21	JAOVZF010000163.1	RiPP-like, NRPS	269655	313831
Region 22	JAOVZF010000151.1	LAP, APE	113265	184966
Region 23	JAOVZF010000164.1	Class-I lanthipeptide, RiPP-like	105469	134267
Region 24	JAOVZF010000162.1	Class-I lanthipeptide	312593	342102

Table S4. RiPP BGCs revealed by RiPPMiner.

Cluster	Locus (contigs)	RiPP class	From	To
Cluster 1	JAOVZF010000146.1	Glycocin	115465	174581
Cluster 2	JAOVZF010000146.1	Head-to-tail cyclized	178690	235768
Cluster 3	JAOVZF010000151.1	Class-I lanthipeptide	85529	149744
Cluster 4	JAOVZF010000163.1	LAP	86181	174179
Cluster 5	JAOVZF010000164.1	Class-I lanthipeptide	309536	377957

Table S5. COG classification of the protein-coding genes of five strains in the family Nannocystaceae.

COG functional categories	Number of genes*				
	N. exedens **DSM 71**	*P. sp.* **WMMC 2535**	*"P. miuraensis"* **SMH-27-4**	*E. salina* **DSM 1520**	*P. pacifica* **SIR-1**
A: RNA processing and modification	3	0	3	1	2
C: Energy production and conversion	200	17	186	169	165
D: Cell cycle control, cell division, chromosome partitioning	59	82	85	79	72
E: Amino acid transport and metabolism	166	156	174	162	185
F: Nucleotide transport and metabolism	64	61	63	53	65
G: Carbohydrate transport and metabolism	102	73	98	79	90
H: Coenzyme transport and metabolism	158	154	151	141	164
I: Lipid transport and metabolism	266	252	244	260	256
J: Translation, ribosomal structure and biogenesis	238	237	231	222	215
K: Transcription	442	278	338	257	381
L: Replication, recombination and repair	147	137	137	138	118
M: Cell wall/membrane/envelope biogenesis	246	207	275	224	215
N: Cell motility	20	21	21	22	18
O: Posttranslational modification, protein turnover, chaperones	255	229	263	237	243
P: Inorganic ion transport and metabolism	139	125	121	122	117
Q: Secondary metabolites biosynthesis, transport and catabolism	90	60	63	81	74
T: Signal transduction mechanisms	599	489	635	474	490
U: Intracellular trafficking, secretion, and vesicular transport	54	60	68	56	45
V: Defense mechanisms	175	117	143	135	112
W: Extracellular structures	14	13	21	15	13
X: Mobilome: prophages, transposons	105	35	31	24	35
Z: Cytoskeleton	7	17	11	16	8
R: General function prediction only	387	354	461	362	374
S: Function unknown	84	61	71	63	61

*The numbers of genes were counted using in-house Python script and rounded to intigers.

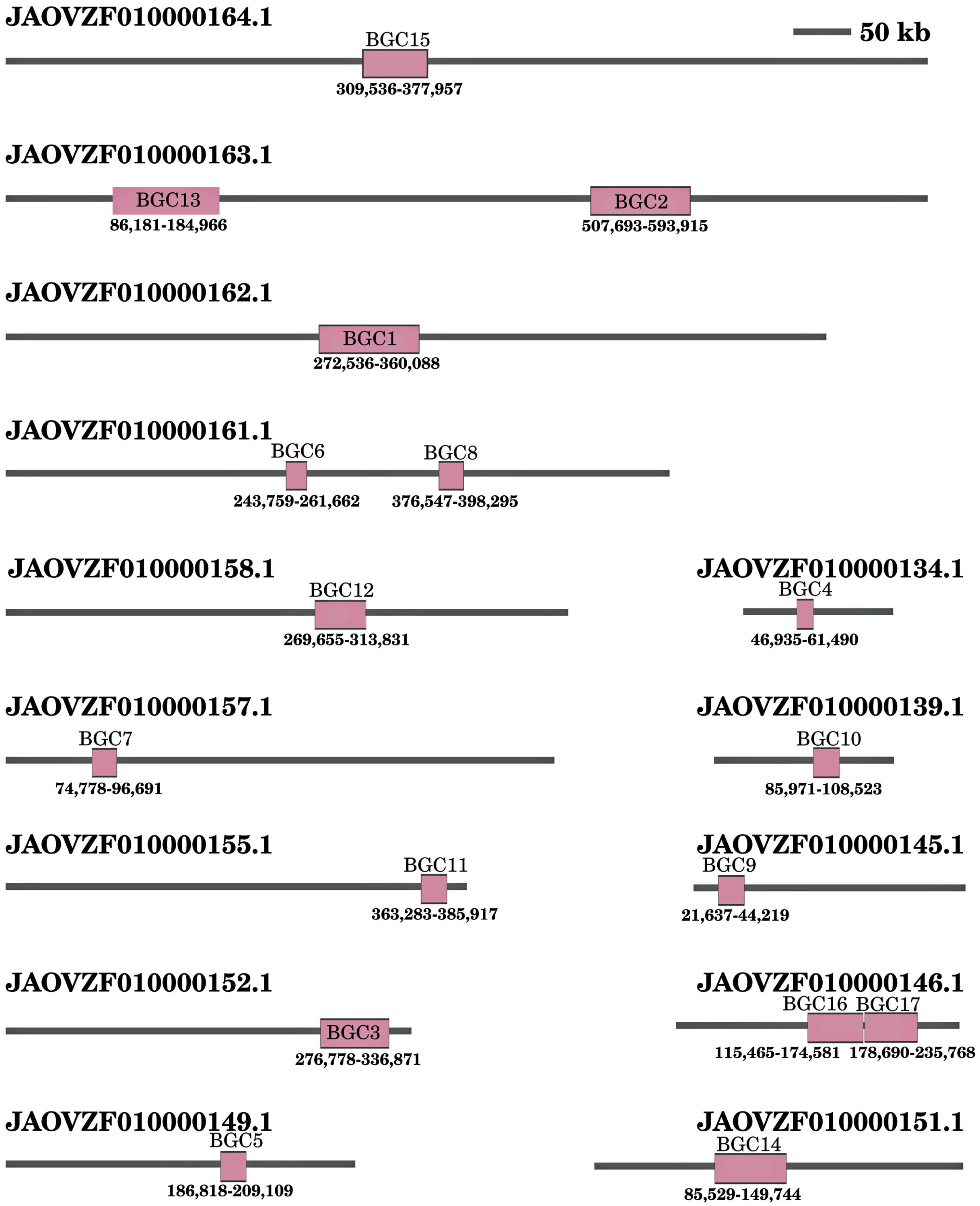

Figure S1. Locations of BGCs.

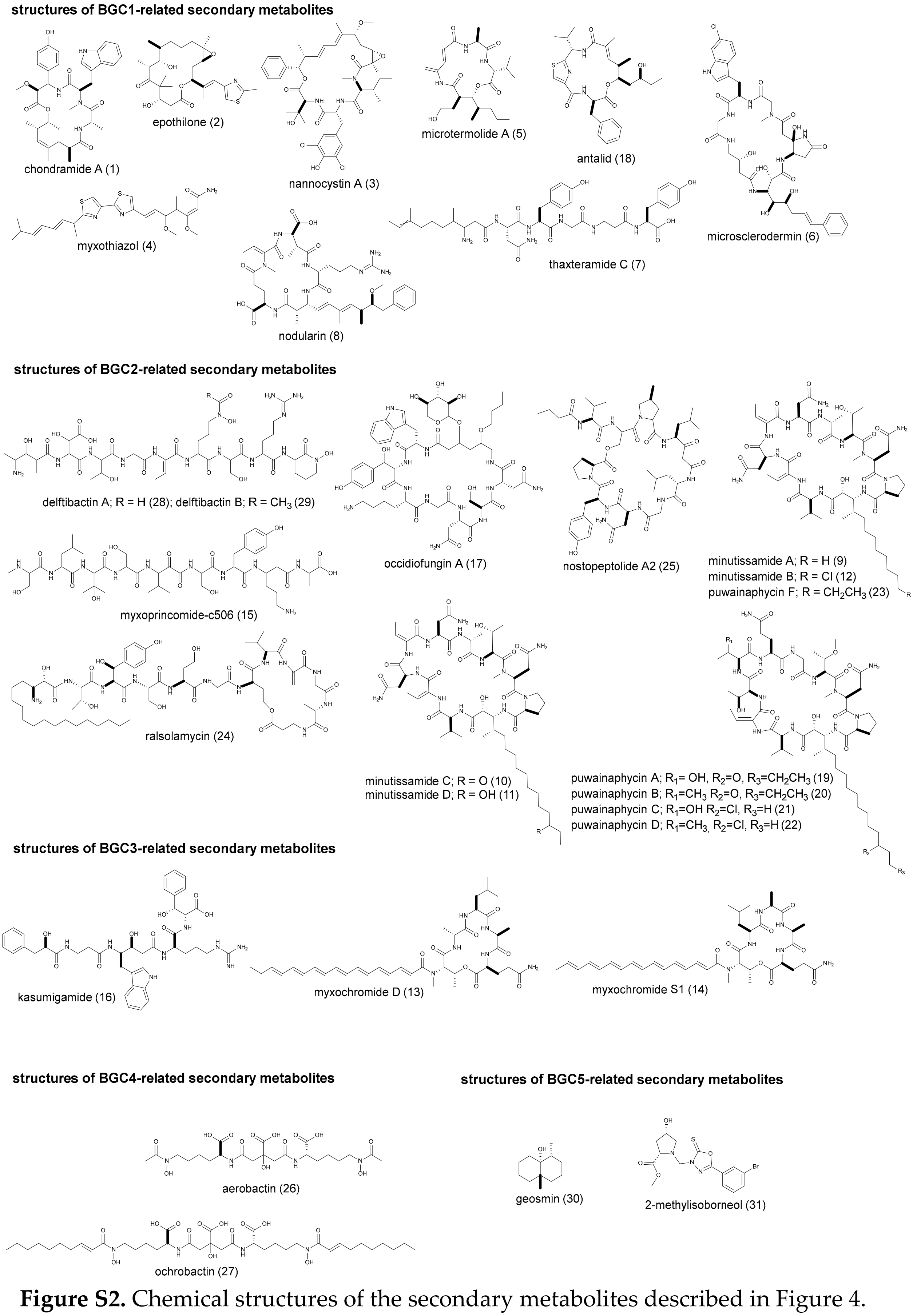

Figure S2. Chemical structures of the secondary metabolites described in Figure 4.

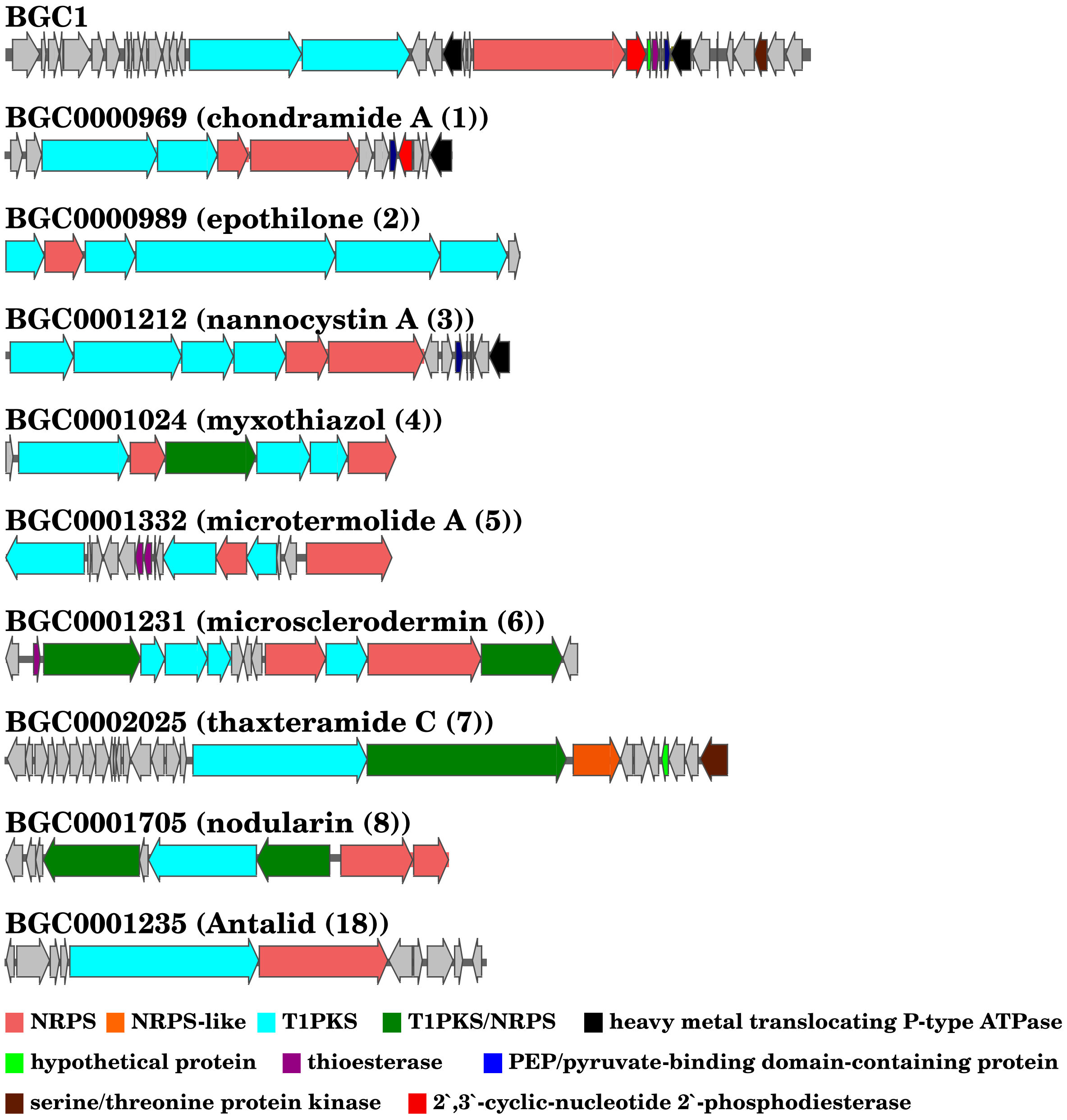

Figure S3. Organizations of BGC1 and its related BGCs of other species. PKS and NRPS related genes (NRPS, NRPS-like, T1PKS, and T1PKS/NRPS) are colored by their types. The other colored genes (except grey) are based on blastp results (e-value ≤10^{-10}, identity ≥30%).

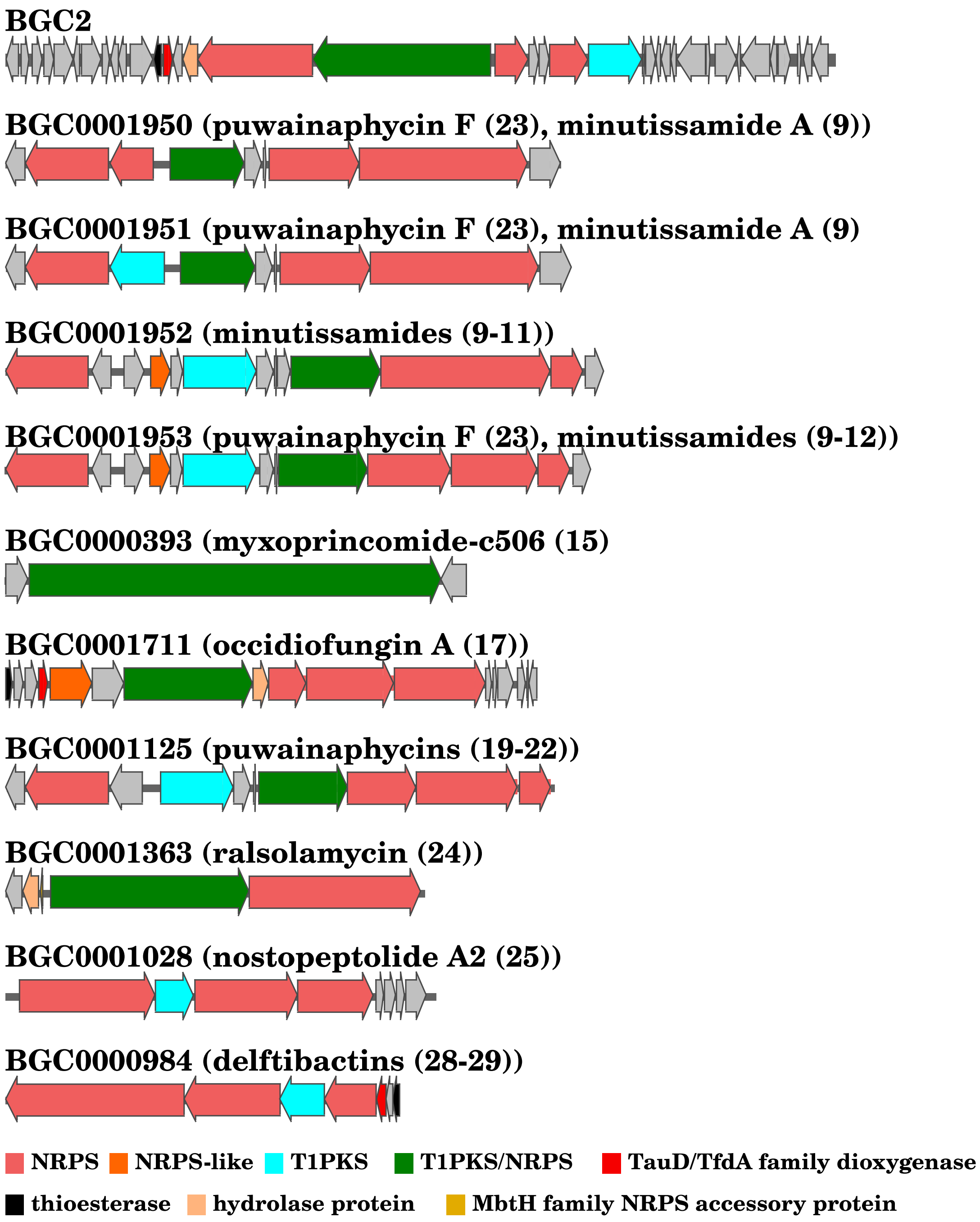

Figure S4. Organizations of BGC2 and its related BGCs of other species. PKS and NRPS related genes (NRPS, NRPS-like, T1PKS, and T1PKS/NRPS) are colored by their types. The other colored genes (except grey) are based on blastp results (e-value $\leq 10^{-10}$, identity $\geq$30%).

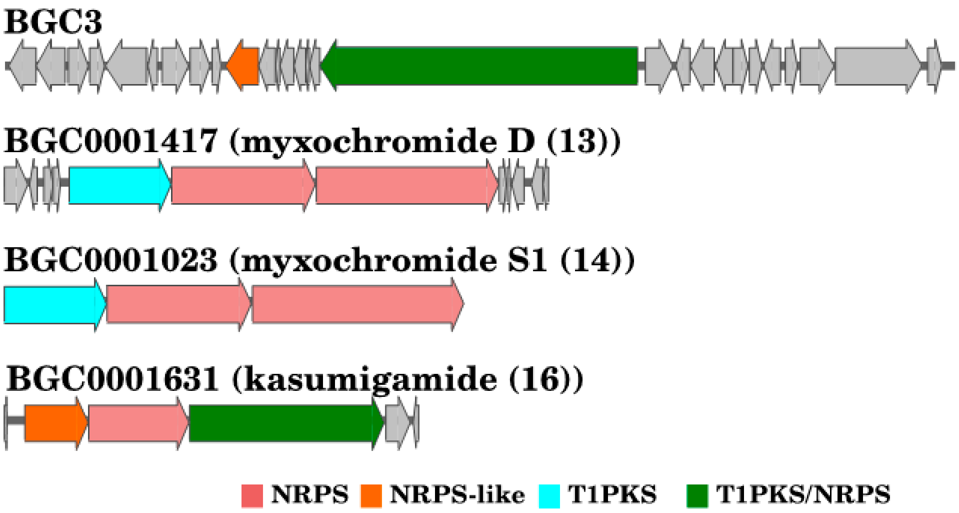

Figure S5. Organizations of BGC3 and its related BGCs of other species. PKS and NRPS related genes (NRPS, NRPS-like, T1PKS, and T1PKS/NRPS) are colored by their types. The other colored genes (except grey) are based on blastp results (e-value ≤10^{-10}, identity ≥30%).

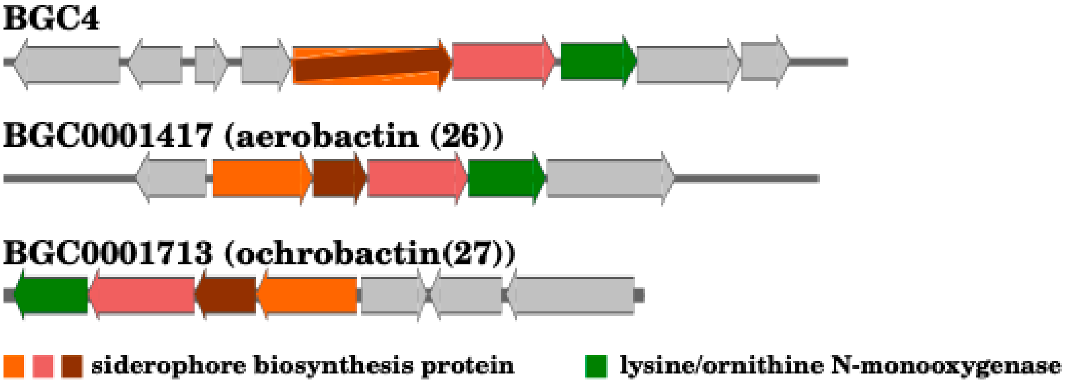

Figure S6. Organizations of BGC4 and its related BGCs of other species. Genes are colored (except grey) based on blastp results (e-value ≤10^{-10}, identity ≥30%).

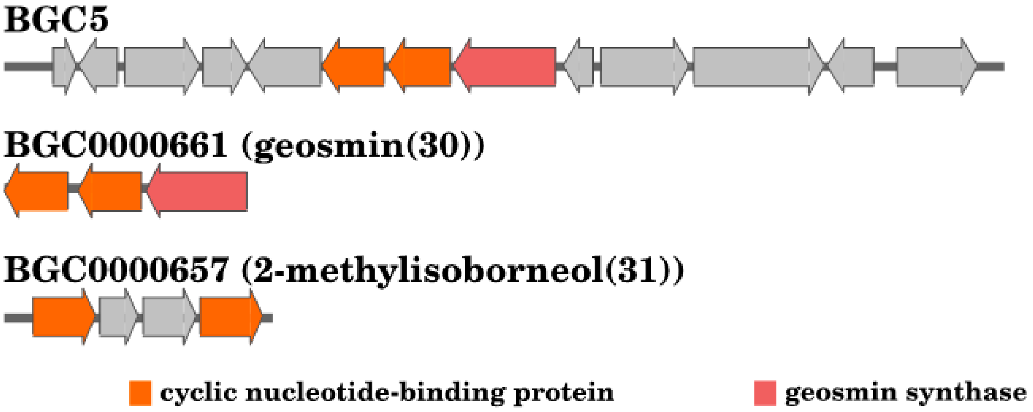

Figure S7. Organizations of BGC6 and its related BGCs of other species. Genes are colored (except grey) based on blastp results (e-value ≤10^{-10}, identity ≥30%).

Reference S1 (a part of ref. 14, p86-87)

Description of Paraliomyxa gen. nov.

Paraliomyxa [Paralio.myxa. Gr. adj. paralio growing or living in costal environment, Gr. Fem. N. myxa slime; N. L. fem. N. Paraliomyxa slim of costal habitat, that means coastal myxobacteria]. Cells are rod-shaped, move by gliding and form swarms on agar media. Fruiting body (FB)-like structures and myxospore-like optically refractive cells are formed. Mesophilic bacterium which grows between 18-40 °C. Requires lower concentration of NaCl than the average seawater level for the growth. Grows at neutral pH range. The fatty acid profile is characterized by major amount of BCFAs and minor amount of SCFAs, when cultured in N0.5-S20-10 liquid medium. PUFAs are not found among cellular FAs. The major cellular quinone is MK-8. The DNA G + C content of the type strain of the species is 69-70 mol %. As shown by 16S rRNA gene sequence analysis, the genus belongs to the suborder Nannocystineae, the order Myxococcales, the class Deltaproteobacteria. The phylogenetic position of the genus is distantly related to the genus Enhygromyxa and the genus Plesiocystis. The type species is Paraliomyxa miuraensis.

Description of Paraliomyxa miuraensis sp. nov.

Cells are gliding rods, measuring 0.5-0.8 x 2.0-5.0 μm. In addition to the characteristics reported above in the genus description, type strain was found to produce the novel antifungal depsipeptides: miuraenamides. The strain required very low concentration of NaCl for the growth. No distinct fruiting body was observed for the strain. The major cellular quinone was MK-8, and the major cellular fatty acid components were iso acids (iso-C15:0 and iso-C17:0). Long chain PUFAs (poly unsaturated fatty acids) and hydroxy fatty acids were not detected. Caseinase was detected but amylase was not. On the basis of phylogenetic, chemotaxonomic and physiological data, Paraliomyxa miuraensis gen. nov., sp. nov. is proposed. Mesophilic bacterium, no growth occurs above 40 °C or below 15 °C. NaCl concentration range for the growth is between 0.0-3.0% (w/v), with an optimum at 0.5-1.5%. No growth in higher NaCl concentration than 3.5% (w/v). It requires both Mg2+ and Ca2+ for the growth, and does not grow in VY/2 agar for terrestrial myxobacteria. The optimum growth pH range is pH 7.0-7.5. No growth below pH 5.5 or above pH 8.5. Oxidase was positive but catalase was weak or negative. Colonies are Congo-red-negative. Aerobic. It decomposes live E. coli cells, but does not lyse autoclaved yeast cells. Caseinase and gelatinase are positive. The strain is rather oligotrophic, and grows in CY/6-S75-15 medium but not in CY/3-S75-15. Does not utilize nitrate as nitrogen source. Amylase is negative. Does not degrade filter paper. It sometimes cleaves the agar gel matrix within the swarm area, but does not completely liquify agar gel. Tween 80 lipase and DNAase are negative. The major cellular fatty acids are iso-C15:0 (29.5%) and iso-C17:0 (33.5%). Iso-C15:1 (8.0%) and iso-C16:0 (3.3%) were also detected. It lacks anteiso-acids, long chain PUFAs and hydroxylated acids. The type strain is SMH-27-4T.
